# ﻿A new combination for a neglected member of *Linaria* subsect. *Versicolores* (Plantaginaceae, Antirrhineae) endemic to the Algarve, Portugal

**DOI:** 10.3897/phytokeys.243.122788

**Published:** 2024-06-19

**Authors:** João Farminhão, André Carapeto

**Affiliations:** 1 Jardim Botânico da Universidade de Coimbra, Calçada Martim de Freitas, 3000-456 Coimbra, Portugal Sociedade Portuguesa de Botânica Alverca do Ribatejo Portugal; 2 Centre for Functional Ecology, Laboratório Associado TERRA, Departamento de Ciências da Vida, Universidade de Coimbra, Calçada Martim de Freitas, 3000-456, Coimbra, Portugal Jardim Botânico da Universidade de Coimbra Coimbra Portugal; 3 Sociedade Portuguesa de Botânica, Travessa do Jardim n.º 3 2615-018 A-dos-Potes, Alverca do Ribatejo, Portugal Universidade de Coimbra Coimbra Portugal

**Keywords:** Flower colour, Iberian Peninsula, iNaturalist, lectotypification, Mediterranean flora, plant taxonomy, endemic to the Algarve, stone pine, toadflax

## Abstract

*Linariabimaculata***comb. et stat. nov.**, from the overlooked Central Algarve plant endemism centre, is here lectotypified and redescribed as a full species based on Linariaviscosavar.bimaculata, which was historically misidentified under allopatric *L.spartea* and *L.viscosa*. Traditional herbarium taxonomy and citizen science observations were combined to document the geographical range of the four species of Linariasubsect.Versicolores in the Algarve and amend an identification key for the Iberian clade of this subsection. Geographical patterns and morphological similarity suggest a sister relationship between *L.bimaculata* and *L.algarviana*, unveiling a new possible example of parallel speciation linked to a purple to yellow shift in corolla colour. Besides the yellow flowers, *L.bimaculata* differs from *L.algarviana* in the more elongate fertile stems and the invariably erect-patent corolla tube. It is assessed as Vulnerable (VU) according to the IUCN Categories and Criteria.

## ﻿Introduction

The Iberian clade of Linariasubsect.Versicolores (Benth.) Wettst. comprises eight currently accepted taxa, being keyed out from the other Iberian congeners by their distinctly bifid stigma (Fernández-Mazuecos et al. 2018a). Corolla colour divergence between parapatric or sympatric sister species and convergence in allopatric species are a hallmark of this clade (Fernández-Mazuecos et al. 2018b). As an example, and doing justice to this subsection’s name (i.e., colour-changing), a colour shift of the corolla from purple to yellow has occurred independently twice in the Iberian Peninsula, namely in the common ancestor of *L.viscosa* (L.) Chaz. and *L.onubensis* Pau in southwestern Iberia, and in the common ancestor of *L.spartea* (L.) Chaz. and *L.incarnata* (Vent.) Spreng. in central Iberia (Fernández-Mazuecos et al. 2018b). Here, we resuscitate an overlooked taxon from coastal central Algarve, in southernmost Portugal, which has been confused with yellow-flowered *L.viscosa* and *L.spartea*. It probably represents an additional case of corolla colour convergence and a purple-yellow shift coupled with speciation, considering that we here infer *L.algarviana* Chav. as its hypothetical sister.

*Linariaviscosa*, the type of subsection Versicolores, is endemic to Southern and Eastern Spain, while *Linariaspartea* is widespread in Iberia and southwestern France (Fernández-Mazuecos et al. 2018a). The latter is renowned for accommodating significant morphological variation (Sutton 1988, Sáez 2009), which is reflected in the existence of 10 heterotypic synonyms (POWO 2024), including *Linariapraecox* Hoffmanns. & Link (Hoffmannsegg and Link 1811: 234). The type of *L.praecox* was collected in the "*champs sablonneux de l’Algarve*", with no mention of a precise locality, certainly during the voyage of Johan Centurius von Hoffmannsegg and Johann Heinrich Friedrich Link to the Algarve in February–March 1799. Most of the type material was probably included in Link’s personal herbarium, which was destroyed during the bombing of the Berlin Herbarium in 1943 (Medina and Aedo 2022). The surviving original material at Berlin, consisting of a single flower (B-W11283-010), which was part of Hoffmannsegg’s herbarium bequeathed to Willdenow, and the associated colour plate in the *Flore Portugaise* (Hoffmannsegg and Link 1811: t37), agree well with *L.spartea*. This taxon is still namely present in the sand fields west from the mouth of the Guadiana (Domingues de Almeida et al. 2024) together with plants tentatively identified as *L.viscosa* (Domingues de Almeida et al. 2024). This area was visited by the two German botanists during their sojourn in Vila Real de Santo António (Gomes Oliveira 2015). After the original description, the name *L.praecox* – transferred to *Antirrhinum* L. by Brotero (1828) – and its recombination Linariasparteavar.praecox (Hoffmanns. & Link) Willk. & Lange, were first applied to plants collected around Faro, in central Algarve (Willkomm and Lange 1861; Henriques 1889), but later the use of this name – including the synonym Linariajunceavar.praecox Hoffmanns. & Link ex Samp. (Sampaio 1913: 111) – changed to encompass other early-blooming populations of *L.spartea* in Portugal (e.g., Coutinho 1906; Sampaio 1946; Viano 1973, 1978).

Plants from a narrow coastal strip centred in Faro, between Albufeira and Olhão, were later described as Linariaviscosavar.bimaculata Cout. (1916:10) based on the presence of two conspicuous longitudinal brownish red stripes on the throat of the corolla, and later accepted under this name by Coutinho (1935, 1939) and Viano (1973, 1976, 1978). These plants may correspond to what Hoffmannsegg and Link (1811: 256) designated as “Linariabipunctatavar.bipunctata”, collected in the “(…) *Algarve, aux lieux sablonneux entre Villanova et Lagôa*”, in February–March 1799, described as having the “*Palais à 2 points pourpres-noirâtres*”. Unfortunately, no material of Linariabipunctatavar.bipunctata sensu Hoffmannsegg & Link has survived at the Berlin Herbarium. More recently, Sutton (1988: 436), while advocating for further studies, reassigned plants from the vicinity of Faro to *L.praecox*, including L.v.var.bimaculata, describing them as somewhat intermediate between *L.spartea* and *L.viscosa*, and characterised by a well-developed basal rosette of sterile shoots, relatively small leaves, a sparsely glandular inflorescence and small flowers. Yet, the latest monographic works on Linariasubsect.Versicolores from the Iberian Peninsula (Sáez 2009; Fernández-Mazuecos et al. 2018a) did not recognise this taxon, including it implicitly within *L.spartea*. As a result of all the different views on its taxonomic status, plants ascribable to Linariaviscosavar.bimaculata were also identified as *L.spartea* (Costa et al. 1996; Pereira et al. 2007) or *L.viscosa* in floristic studies (Carapeto 2020), and correspond to most records of *L.spartea* in the Algarve uploaded on iNaturalist between 2015 and 2023, one of them (i.e., 1694064) being selected as the thumbnail image for *L.spartea*. However, these plants differ in multiple morphological characters from *L.spartea* and *L.viscosa*, or from any other *Linaria*, being most similar to *L.algarviana*, endemic to western Algarve, with which they share the typically decumbent fertile stems and broadly ovate, divergent and slightly reflexed upper petals. Accordingly, we here raise L.v.var.bimaculata to full species and provide an updated taxonomic account for this narrow endemic, including a risk of extinction assessment, together with an amended key to the Iberian clade of Linariasubsect.Versicolores.

## ﻿Material and methods

We applied standard herbarium practices to study the variation of plants ascribable to Linariasubsect.Versicolores in the Algarve, namely material referable to *L.algarviana*, *L.spartea* and *L.viscosa*, including L.viscosavar.bimaculata, at ALGU, COI, LISE, LISI, LISU, PO (incl. PO-GS) and MA (acronyms following Thiers, continuously updated). Additionally, we examined all scans of Linariasubsect.Versicolores from southwestern Iberia and northwestern Africa published on GBIF (2024), including those facilitated at the online catalogue of P (https://science.mnhn.fr/institution/mnhn/collection/p/item/search). This enabled us to locate additional specimens of L.viscosavar.bimaculata at BR, P, W and WAG. No records of L.viscosavar.bimaculata were found outside the Algarve. Herbarium specimens of L.viscosavar.bimaculata were photographed with a scale and, subsequently, the acquired images were utilised to score multiple quantitative characters to the nearest 0.1 mm with the “Measure” tool from ImageJ v.1.52.d. Specimens of L.viscosavar.bimaculata were described following recent taxonomic references on Iberian *Linaria* (Sáez 2009; Fernández-Mazuecos et al. 2018a; Blanca et al. 2023), and the chromosome number for this taxon was retrieved from Viano (1973). Seeds were examined under an Emspira 3 digital microscope (Leica Mycrosystems) and photographed with Application Suite X (LAS X). The key to the Iberian clade of L.subsect.Versicolores by Fernández-Mazuecos et al. (2018a) was amended, from couplet number 9, to accommodate the newly reappraised taxon.

We combined herbarium taxonomy with a review of iNaturalist (2024) records of *Linaria* in the Algarve, uploaded until January 31, 2024. A total of 78 occurrences of L.viscosavar.bimaculata were identified (Appendix 1). No observations of similar plants were found in other botanical provinces of Portugal, Spain or Morocco.

Herbarium and iNaturalist records, together with occurrence data available through Flora-On (Pereira et al. 2016), were used to plot the distribution of the different taxa of Linariasubsect.Versicolores (viz. *L.algarviana*, *L.spartea*, *L.viscosa* and L.viscosavar.bimaculata) in the Algarve, on ArcGis 10.4. A risk of extinction assessment was prepared following Carapeto et al. (2020) and using the IUCN Red List guidelines (IUCN Standards and Petitions Committee 2022). Extent of Occurrence (EOO) and Area of Occupancy (AOO) were calculated using GeoCAT (Bachman et al. 2011).

## ﻿Taxonomic treatment

### ﻿Identification key (amendment to Fernández-Mazuecos et al. 2018a)

**Table:** 

9a	Corolla tube erect-patent; throat with 2 longitudinal brownish red to blackish brown stripes	***L.bimaculata***
9b	Corolla tube erect; throat with no markings or with multiple darker veins	**10**
10a	Inflorescence predominantly lax, glabrous, sparsely glandular-pubescent or densely glandular-pubescent, fruit pedicels porrect	***L.spartea***
10b	Inflorescence predominantly dense, corymbiform at anthesis, generally densely glandular-pubescent, fruit pedicels appressed	**10**
11a	Pedicels ± adnate in his basal part to the inflorescence axis; calyx lobes 0.4–0.9 mm wide	***L.salzmannii***
11b	Pedicels not adnate to the inflorescence axis; calyx lobes 0.9–1.8 mm wide	***L.viscosa***

#### ﻿ Linaria bimaculata

Taxon classificationPlantaeLamialesPlantaginaceae

(Cout.) Farminhão & Carapeto, comb. et stat. nov.

D52E616D-E74F-5F4C-8264-B39D9D732932

urn:lsid:ipni.org:names:77343737-1

Figs 1 , 2

##### Basionym.

Linariaviscosavar.bimaculata Cout., Notas Fl. Portugal III: 10 (1916).

##### Type.

**Figure 1.**
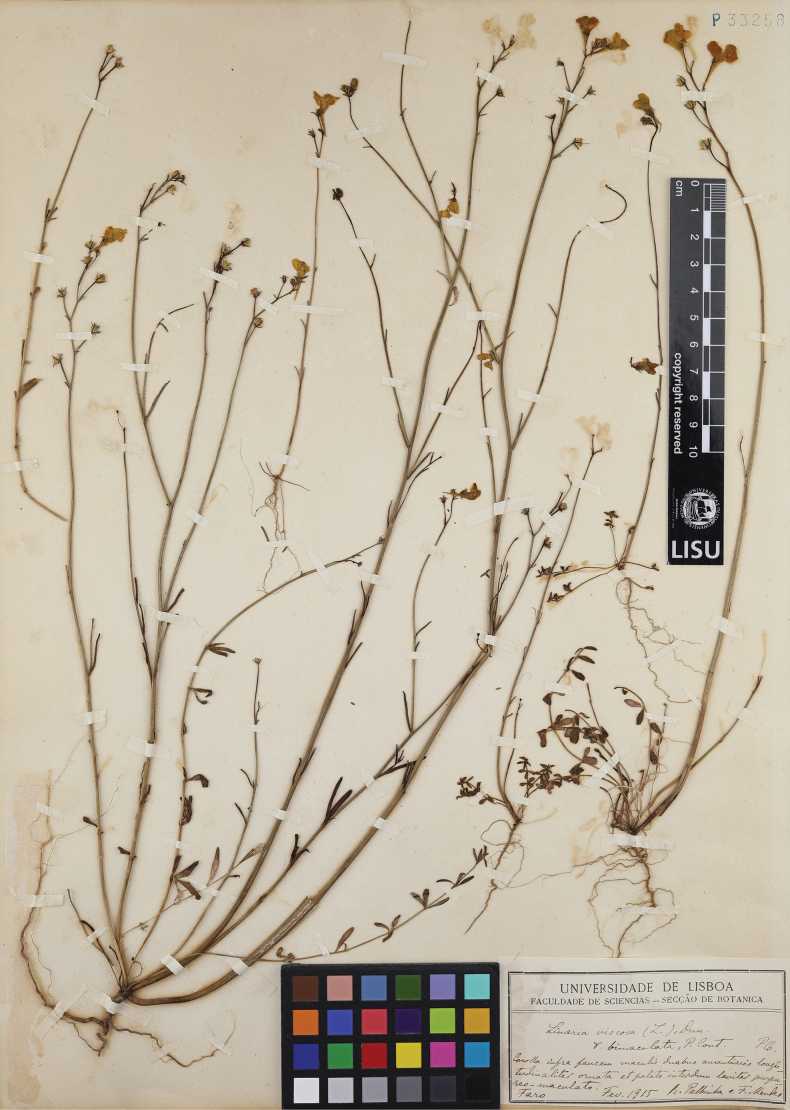
Lectotype of *Linariabimaculata* (Cout.) Farminhão & Carapeto (*Palhinha & Mendes s.n.*, LISU33258).

Portugal. Algarve: Faro, February 1915, *R. Palhinha & F. Mendes s.n.* (***lectotype*** LISU [LISU33258!], designated here, Fig. 1; isolectotypes LISE [LISE83092!], PO [PO20408!]).

##### Description.

Annual herb; somewhat glaucous, glabrous, except for glandular-pubescent inflorescence, hairs 0.4–0.5 mm. Fertile stems 1–3(–8), (4.6–)18–33.3(–41.8) cm long, decumbent to ascending or erect, simple or 2–4(–10)-branched; sterile stems (1–)4–10(–29), (1.6–)3.9–8.5(13.6) cm long, prostrate to decumbent, simple, sometimes forming a dense rosette. Leaves of fertile stems (3.3–)6–13.9(–30.6) × (0.4–)0.7–1.3(–2.5) mm, linear, flat to revolute, obtuse to ± acute, alternate, sometimes the intermediate in whorls of 3; leaves of sterile stems (1.8–)3.4–8.6(–17.8) × (0.3–)1–2.1(–3.1) mm, linear to ovate, flat, in whorls of 3(–4). Inflorescence racemose, rachis up to (2–)2.6–4.3(–6.2) cm long in fruit, green or red, with (1–)4–7(–14) flowers, lax in flower and fruit. Bracts (2–)2.3–2.8(–3.4) × 0.2–0.4 mm, linear, acute, glabrous or glandular. Pedicels (3.7–)5.2–7.8(–9.6) mm long in flower, (3.4–)6–9.4(–12.9) mm long in fruit, erect, not adnate to the inflorescence axis, red. Calyx lobes (2.2–)2.3–2.9(–3) × (0.4–)0.7–0.9(–1.1) mm in flower and (2.4–)2.9–3.5(–4.1) × (0.6–)0.8–1.1 mm in fruit, subequal, glandular-pubescent, linear-lanceolate, acute, green sometimes red-tinged with whitish scarious margin. Corolla personate, spurred, (13.1–)14.8–17.6(–19.8) mm long, deep yellow with 2 longitudinal brownish red to blackish brown stripes on the throat, and an orangey palate, sometimes with brownish red spots or reticulate markings, without conspicuous dark veins; tube (1.9–)2.4–3.2(–3.7) mm broad in dorsiventral section, erect-patent; upper petals broadly ovate, divergent, slightly reflexed; spur (5.7–)7.9–9.6(–10.9) × 1–1.6(–1.9) mm (the width measured at the base), straight or slightly curved, equalling to slightly shorter than the rest of the corolla. Capsule (2.1–)2.4–3.1 × (1.6–)2–2.8 mm, globose, glabrous, loculi equal; style 2.1–2.5(–3.2) mm long, persistent, bifid at apex. Seeds (0.5–)0.6–0.7 × 0.4–0.5(–0.6) mm, wingless, pyriform-triquetrous, transversely ridged, alveolate, black. 2*n* = 12.

##### Habitat and distribution.

*Linariabimaculata* is endemic to coastal central Algarve, from Galé (Albufeira) in the west to Pinheiro (Tavira) to the east, up to 50 m a.s.l (Fig. 3). Its distribution is centred on the Plio-Pleistocene medium to coarse grain siliceous sands and gravels of the Ludo Formation (Moura and Boski 1994) in central Algarve. It occurs mostly on clearings and at fringe of *Pinuspinea* L. and *P.pinaster* Aiton woods and scrubland with Ulexargenteussubsp.subsericeus (Cout.) Rothm., *Stauracanthus* spp. and *Cistus* spp. (Costa et al. 1996; Pereira et al. 2007; Carapeto 2020). *Linariabimaculata* integrates psammophilic communities protected from the direct influence of sea spray, where characteristic species include mostly ephemeral annuals, namely *Tuberariaguttata* (L.) Fourr., *Tolpisbarbata* (L.) Gaertn., *Brizamaxima* L., *Silenescabriflora* Brot., *Plantagobellardii* All., *Rumexbucephalophorus* L., *Marcuskochia-triloba* (L.) Al-Shehbaz and *Ornithopuspinnatus* (Mill.) Druce, described as the association *Tolpidobarbatae*-*Tuberarietumbupleurifoliae*, endemic to the Algarve (Costa et al. 1996).

##### Phenology.

Flowering from December to June (September), peaking between January and April. Fruits develop mostly from March to June.

##### Conservation assessment.

*Linariabimaculata* presents a restricted distribution range in coastal central Algarve. The EOO comprises 271.8 km^2^ and the AOO is 152 km^2^. The population faces several threats, including urban and touristic development, agricultural intensification, and the expansion of alien plants and nitrophilous communities as result of human disturbance. These ongoing threats are responsible for continued declines in the area and quality of the habitat. A continued decline in population size and AOO can also be inferred from the habitat loss and from disappearance from historical collection sites (e.g. near Faro). Considering the urban/touristic expansion within its distribution range as the main cause of habitat loss and fragmentation, only eight locations are identified, therefore this plant is assessed as Vulnerable, fulfilling the criteria B1ab(ii,iii,v)+2ab(ii,iii,v).

##### Notes.

**Figure 2.**
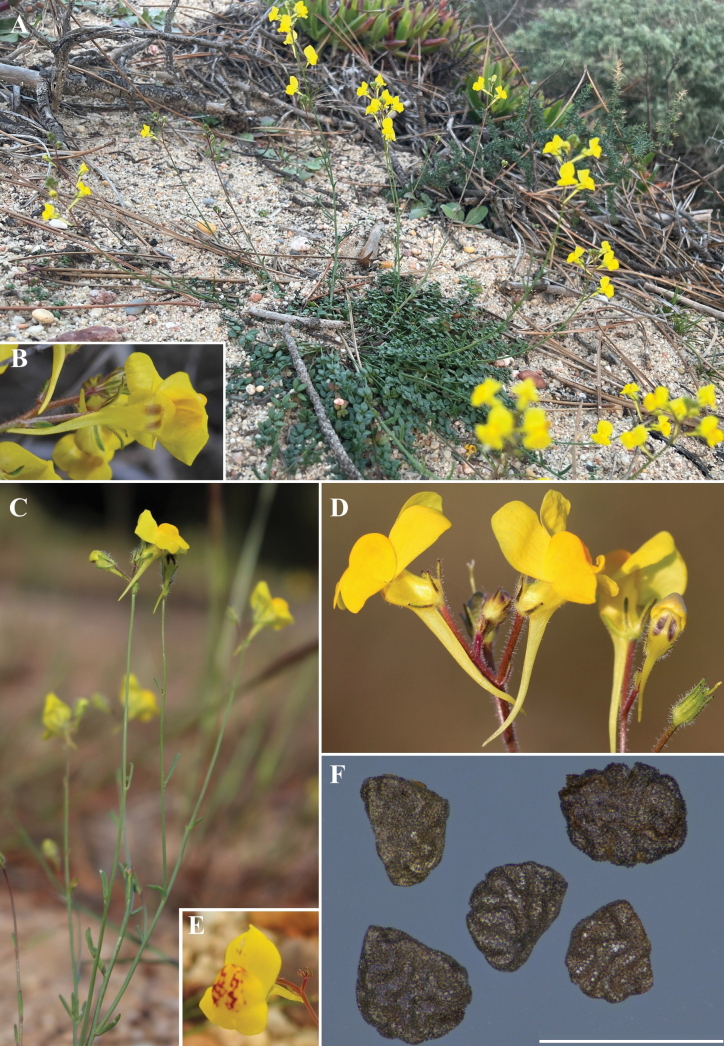
Overview of *Linariabimaculata* (Cout.) Farminhão & Carapeto **A** habit (ascending form) and habitat **B** underside of flower with visible stripes **C** habit (erect form) D flowers with erect-patent corolla tube, immaculate palate and stripes visible on the underside of flower bud **E** flower with reticulate palate **F** seeds (*A. Moller s.n.*, COI), scale bar 1 mm. Photos by M. Hansch (**A**), D. Frade (**B, E**), V. Dvořák (**C**), J. Neiva (**D**) and A. Coelho (**F**).

Coutinho (1916) does not cite any type material in the protologue of Linariaviscosavar.bimaculata, but only one gathering, *R. Palhinha & F. Mendes s.n.* from Faro, can be regarded as original material, with duplicates at LISE, LISU and PO. The duplicate at LISU is labelled with a Latin diagnosis in Coutinho’s handwriting matching the protologue in Portuguese, therefore being here selected as the lectotype. Besides the type collection, the only other specimen determined as Linariaviscosavar.bimaculata by Coutinho, *R. Palhinha & F. Mendes s.n.* (LISU) from Ilha das Lebres (Olhão), was only collected after the original publication of this taxon. The reticulate pattern of the palate (Fig. 2E) described in the type material (Fig. 1) is absent from most individuals observed in the field, which present an immaculate palate, being absent altogether in some populations. The apparent absence of spatial structure of this trait (i.e.reticulate vs immaculate palate) suggests it is best interpreted as polymorphism.

**Figure 3.**
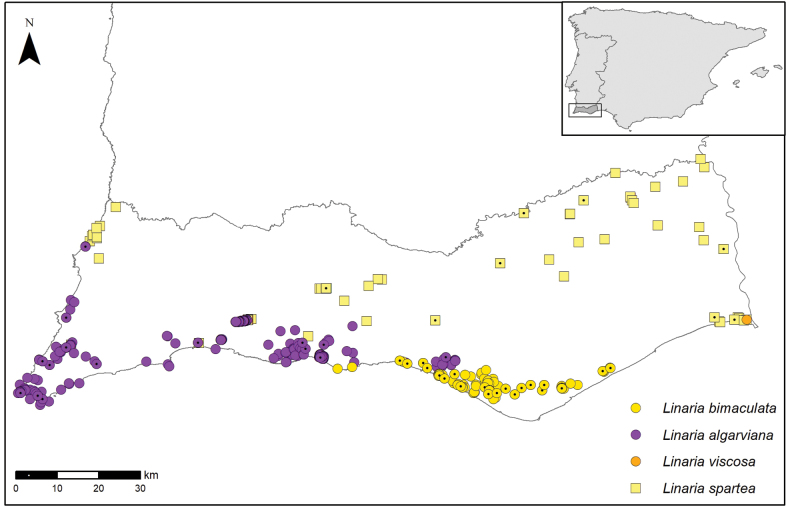
Distribution of *Linariaalgarviana* Chav., *Linariabimaculata* (Cout.) Farminhão & Carapeto, *Linariaspartea* (L.) Chaz. and *Linariaviscosa* (L.) Chaz. in the Algarve, in southwesternmost Iberia. Plain symbols indicate observation records and dotted symbols indicate herbarium specimens.

**Figure 4.**
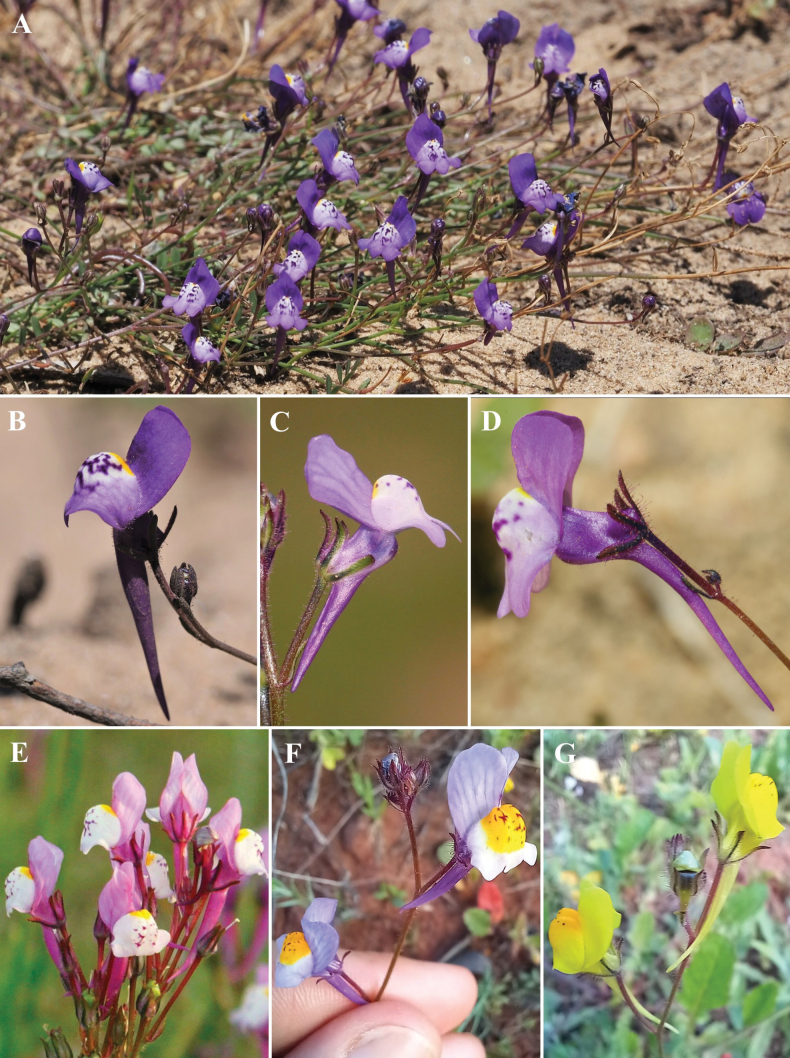
Overview of *Linariaalgarviana***A** habit **B** dark purple morph (Aljezur) typical of the westernmost populations **C** light purple morph (Loulé) **D** flower with erect-patent corolla tube (Loulé) **E** pink morph **F** bicolorous morph (Portimão) **G** yellow morph (Portimão). Photos by V. Achterberg (**A, B**), J. Neiva (**C**), J.T. Tavares (**D**), A.J. Pereira (**E**) and S. Lobo Dias (**F, G**).

*Linariaalgarviana*, hypothetically the closest relative of *L.bimaculata* based on flower and habit similarity, presents multiple, although rare, colour morphs (Fig. 4), which are here illustrated for the first time. Darker flowers (Fig. 4A, B) occur on the western part of its range (Aljezur, Vila do Bispo). Flowers with an erect-patent corolla tube, similar to those of *L.bimaculata*, occur sporadically towards the eastern part of its range (Loulé). Also, there are yellow-flowered individuals of *L.algarviana* (Fig. 4G), that can be distinguished from *L.bimaculata* by the erect corolla tube and the paler throat stripes. This colour polymorphism, involving purple, yellow and bicolour morphs is similar to the one reported in *Linariasalzmannii* Boiss., another Iberian species of Linariasubsect.Versicolores (Fernández-Mazuecos et al. 2018a).

Differences between *L.bimaculata* and other Linariasubsect.Versicolores present in the Algarve are summarised in Table 1. *Linariabimaculata* differs from *L.algarviana* in the more elongate fertile stems and the yellow flowers, with an invariably erect-patent corolla tube.

**Table 1.** Synopsis of key traits in Linariasubsect.Versicolores present in the Algarve.

	*L.bimaculata*	*L.algarviana*	*L.viscosa*	*L.spartea*
Fertile stem length (cm)	5–42	14–25	5–80	15–55
Fertile stem position	decumbent to ascending or erect	decumbent to ascending or erect	erect or sometimes ascending	erect or sometimes ascending, rarely decumbent
Inflorescence	lax, densely glandular-pubescent	lax, densely glandular-pubescent	dense, densely glandular-pubescent	lax, sparsely to densely glandular-pubescent
Corolla tube position	erect-patent	erect, rarely erect-patent	erect	erect
Corolla colour (typical)	deep yellow with 2 longitudinal brownish red stripes on the throat, and an orangey palate, sometimes with brownish red spots or reticulate markings	violet-purple, the palate whitish with yellow spot and usually reticulated with violet	deep yellow	deep yellow
Upper petals	broadly ovate, divergent and slightly reflexed	broadly ovate, divergent and slightly reflexed	ovate, connivent and markedly reflexed	ovate, connivent and markedly reflexed

##### Additional specimens examined.

**Portugal. Algarve**: Albufeira, Pinhal do Concelho, próximo da praia da Falésia, terreno arenoso em pinhal, 25 Feb 1968, *A. Fernandes, J. Paiva & J. Matos 10115* (COI); Albufeira, estrada da Rocha Baixinha, Olhos de Água, 23 Feb 2019, *M.J. Correia s.n.* (ALGU); Loulé, Vilamoura, pinhal em substrato arenoso, 19 Mar 1995, *M.D. Espírito Santo & J.C. Costa s.n.* (LISI); Loulé, Vilamoura, Borjaca [aldeamento de], areias do Pliocénico, sub-bosque de pinhal manso, com *Oxalispes-caprae*, *Malcolmiagracilima*, 9 Feb 1982, *J. Gomes Pedro, A.M. Medeiros & J.P. Simões 22792* (ALGU); Loulé, east of Quarteira, 7 Apr 1992, *F. Billiet 127* (BR); Loulé, praia do Porto Novo, 1 Mar 2008, *M.D. Espírito Santo & R. Caraça s.n.* (LISI); Loulé, foz do Almargem, 18 Feb 2023, *A. Carapeto s.n.* (COI); Loulé, Trafal, 18 Feb 2023, *A. Carapeto s.n.* (COI); Loulé; entre Almancil e Vale de Lobo, pinhal, 19 Mar 1995, *M.D. Espírito Santo & J.C. Costa s.n.* (LISI); Loulé, near Formosa Park Hotel, exist to the beach, pine grove on white and brown-ochre sand dunes with *P.pinea* and *P.pinaster*, 28 Mar 2004, *L.J.G. van der Maesen 7873* (WAG); Loulé, Ancão, solo arenoso sob pinhal, 5 Feb 2000, *J. Rosa Pinto 436/A* (ALGU); Loulé, Ancão, entre Faro e Ferreiras, base de morro areno-calcáreo com pinhal degradado, 10 Mar 1987, *A. Moura 3079* (MA); Loulé, Quinta do Lago, pinhal, 28 Abr 1989, *J.C. Costa s.n.* (LISI); Faro, Ludo, 22 Feb 1986, *J. Rosa Pinto 436* (ALGU); *ibid. loc.*, 22 Feb 2000, *J. Rosa Pinto s.n.* (ALGU); Faro, S. Pedro, Monte Negro, pinhal de pinheiro-manso em solo arenoso, 3 Feb 1988, *J.C. Costa s.n.* (LISI); Faro, cerca de Gambelas, 11 Apr 2017, *P. Escobar García s.n.* (W); Faro, Marchil, a caminho de Armação de Arábia (salinas), pousio, areias, 2 May 1945, *A.R. Pinto da Silva, C. Fontes, M. Myre & B. Rainha 904* (LISE); Faro, Pinhal de Arábia, solo arenoso-argiloso, 13 Mar 1953, *C. Romariz & E.J. Mendes s.n.* (COI, LISE, LISI, LISU); Faro, entre a cidade e a praia, 13 Jun 1961, *J. Malato-Beliz & J.A. Guerra 5099* (MA); Faro, 3 Feb 1846, *H.M. Willkomm 1377* (COI-WILLK, P [P04057111 (specimen on the right), P04057154]); Faro, Champs sablonneux à Faro, 11 Mar 1853, *E. Bourgeau 1975* (COI-WILLK, P [P03440695, P03440739, P03440744, P04057189]); *sin. loc.*, *E. Bourgeau s.n.* (BR); Faro, in siccis/sabulosis collinis Algarbiae prope Faro, May 1847, *F.M. Welwitsch 257* (COI, LISU, P [P03440692 (3 lowermost specimens), P03440734, P03440742, P03440743, P03440745]); Faro, arredores de Faro, Apr 1889, *A. Moller 707* (COI, P [P03950057, P04057181]); Faro, *s.d.*, *G. Sampaio s.n.* (P-GS); Faro, estrada da Senhora da Saúde, Mar 1883, *J.d’A. Guimarães s.n.* (COI); Faro, Santo António do Alto, Mar 1883, *J.d’A. Guimarães s.n.* (COI); Faro, Areal Gordo, Mar 1891, *J. Brandeiro s.n.* (COI); Olhão, Joinal, areias, Jan 1888, *J. Brandeiro s.n.* (COI); Olhão, Ilha das Lebres, Apr 1917, *R. Palhinha & F. Mendes s.n.* (LISU); Olhão, in pinetis siccis, solo arenoso, 3 Feb 1939, *W. Rothmaler 14383* (LISE); Olhão, Belamandil, pinhal, 17 Feb 2019, *M.J. Correia s.n.* (ALGU); Olhão, Quinta de Marim, no solo greso-calcário do pinhal, 23 Feb 1986, *A. Moura 2864* (COI); *ibid. loc.*, 24 May 1986, *A. Moura 3021* (COI, MA); *ibid. loc.*, pinhal em areias, 3 Feb 1988, *J.C. Costa s.n.* (LISI); *ibid. loc.*, 12 Feb 1993, *J.C. Costa s.n.* (LISI); Olhão, cercanias del centro de educación ambiental de Marim, claros de pinar sobre arenas, 11 Apr 2017, *P. Escobar García 1160/2017* (NY [not seen], W); entre Olhão e Tavira, Quintal de P. Pimentel, junto à estrada, 21 Apr 1956, *J. Malato-Beliz 2849* (MA); Olhão [Tavira], Fuzeta, pr. Livramento, 16 Apr 1963, *B. Rainha 6005* (LISE); Tavira, Livramento, 3 Apr 2024, *A. Carapeto s.n.* (COI); Tavira, Pinheiro, 3 Apr 2024, *A. Carapeto s.n.* (COI).

## ﻿Discussion

Although there is overlap among the differential character states (i.e. stem length and position, corolla tube position) of *Linariabimaculata* and other species of Linariasubsect.Versicolores, the combination of a conspicuously striped yellow corolla and a relatively narrow erect-patent tube, not found anywhere else in the Iberian clade of L.subsect.Versicolores, allows for unambiguous identification of *L.bimaculata* both in live and herbarium specimens. This phenotypic singularity, stable in all investigated populations and not found as part of the intraspecific variability of any closely-related taxa, in association with a well-defined geographic range and habitat requirements, support the recognition of *L.bimaculata* as a *bona fide* taxon, and not as a mere morph. The full species status should be molecularly tested to ascertain its position within the least inclusive clade comprehending *L.algarviana* and *L.spartea* (Fernández-Mazuecos et al. 2018a, b). Future DNA sampling should also target plants of *L.bimaculata* with immaculate (Fig. 2D) and reticulate (Fig. 2E) palates, along with key variants of *L.algarviana*, notably plants with erect-patent corolla tubes (Fig. 4D) similar in shape to *L.bimaculata*, to screen for hybridisation events involving *L.algarviana* and explore additional untapped diversity.

With the recognition of *Linariabimaculata*, the Iberian clade of L.sect.Versicolores now includes nine species (viz. *L.algarviana*, *L.becerrae* Blanca, Cueto & J.Fuentes, *L.bimaculata*, *L.clementei* Haens. ex Boiss., *L.incarnata*, *L.onubensis*, *L.salzmannii*, *L.spartea* and *L.viscosa*). The hypothetical sister relationship between *L.bimaculata* and *L.algarviana* is supported by the observation that closely related species in the Iberian clade of Linariasubsect.Versicolores tend to have close geographical ranges and strikingly divergent floral characters, such as corolla colour (Fernández-Mazuecos et al. 2013, 2018b). Distribution of the four taxa in the Algarve (Fig. 3) conforms to the patterns of corolla colour distribution already described in other areas of the Iberian Peninsula and northwestern Africa for L.subsect.Versicolores (Fernández-Mazuecos et al. 2013, Fernández-Mazuecos et al. 2018b), whereby the hypothetical sisters *L.algarviana*/*L.bimaculata* present a purple-yellow divergence in parapatry and the yellow flowers of *L.bimaculata* are convergent to allopatric *L.viscosa* and *L.spartea*. *Linariaalgarviana* sporadically co-occurs with *L.spartea*, but it is never syntopic with *L.bimaculata*, even in areas of close population proximity (e.g. Quarteira). This geographically structured variation is unlike the distribution pattern of purple and yellow morphs of *Linariasalzmannii*, from southeastern Iberia, which occur mixed in the same populations, and therefore do not correspond to *bona fide* taxa (Fernández-Mazuecos et al. 2018a). However, an integrative approach, combining molecular phylogenetics with statistical morphometrics, following previous studies on Linariasubsect.Versicolores (Vigalondo et al. 2015; Fernández-Mazuecos et al. 2018a, b), will be instrumental to confirm the hypothesised close affinity between parapatric *L.bimaculata* and *L.algarviana* inferred from morphology and biogeography, and thus confirm another instance of parallel speciation linked to colour shifts in *Linaria*. Pollinator shifts are probably coupled with the evolution of these purple-yellow species pairs, but observation studies are long overdue to investigate this niche dimension.

The geographically structured variation of corolla colour in *L.algarviana*/*L.bimaculata* is reminiscent of the pattern found in *Linariaamethystea* Hoffmanns. & Link [Linariasect.Diffusae (Benth) Wettst] in Western Portugal, where yellow-flowered L.amethysteasubsp.multipunctata (Brot.) Chatter & D.A. Webb presents a marginal distribution to the more widespread L.amethysteasubsp.amethystea (Blanca et al. 2023). Arguably, the small differences between *L.bimaculata* and *L.algarviana*, the most prominent being corolla colour, could also be accommodated at subspecies level within *L.algarviana*. However, we prefer not to adopt such treatment for three reasons: 1) the exact phylogenetic position of *L.bimaculata* relative to *L.algarviana* is unknown, 2) no subspecies are currently accepted within L.sect.Versicolores (Fernández-Mazuecos et al. 2018a), 3) the empirical and philosophical merits of recognising subspecies are questionable (Burbrink et al. 2022).

*Linariabimaculata* is one of the three angiosperms endemic to the red sandstone derived soils of Central Algarve, which represent an overlooked centre of plant endemism, obscured by recent taxonomic deflation. The other two endemics, *Tuberariamajor* (Willk.) P.Silva & Rozeira (Cistaceae) and *Scillaodorata* Link (Asparagaceae) were reduced to synonyms in the respective generic treatments of *Flora iberica* (Gallego 2005; Almeida da Silva and Crespi 2013), but they are likely best treated as full species in need of renewed conservation attention (Carapeto et al. 2020). The distribution of *L.bimaculata* largely overlaps with that of *Tettigettalnamariae* (Quartau & Boulard, 1995) in the Algarve, a narrow endemic cicada (Nunes et al. 2014). Both species are mostly restricted to stone pine (*Pinuspinea*) coastal woodlands on sands, which have been largely degraded by urban and tourism encroachment in the last decades. This unfavourable conservation scenario is an extra argument not to postpone the recognition of *L.bimaculata* as a distinct taxon, even if future research would support its treatment as a subspecies of another closely-related species. *Linariabimaculata*, as a Vulnerable narrow endemic, should be added to the Portuguese register of classified natural values (*Cadastro Nacional dos Valores Nacionais Classificados*) to ensure its long-term conservation.

Finally, this study also illustrates the potential of citizen science platforms such as iNaturalist to accelerate the pace of taxonomic work in groups in which diagnostic traits, such as colour (Fritz and Ihlow 2022), have been neglected or considered as unreliable in the past, due to poor preservation in natural history collections.

## Supplementary Material

XML Treatment for Linaria bimaculata

## ﻿Additional information

### Author ORCIDs

João Farminhão https://orcid.org/0000-0002-8811-9895

André Carapeto https://orcid.org/0000-0002-2147-688X

### Data availability

All of the data that support the findings of this study are available in the main text.

## ﻿Appendix 1

**List of iNaturalist records of *Linariabimaculata* (Cout.) Farminhão & Carapeto uploaded until January 31, 2024**

https://www.inaturalist.org/observations/1694064; 1694065; 9152467; 15234186; 15234630; 15234663; 20429494; 33551548; 35569286; 37998325; 38132618; 38263852; 39248890; 42943045; 60163625; 66782895; 69008325; 69045725; 69884823; 70056054; 70280873; 70441524; 71180571; 71501177; 102937835; 105059203; 105655088; 105730603; 106400463; 107103481; 107568403; 107653944; 107657034; 107885110; 108528642; 108836648; 111447319; 112011577; 113006724; 117444569; 144378343; 144762023; 144762024; 146982475; 146983199; 147882882; 148209938; 149340056; 149720631; 149971359; 149993357; 150971013; 151540180; 151635619; 152600749; 152659979; 152962235; 153950334; 154956346; 155000038; 155000128; 155000146; 155014589; 155014835, 163207488, 196153706; 196153746; 196153752; 196235055; 196235079; 196235089; 196266826; 196283753; 196431724; 196587295; 197400046; 197717962; 198040564.

## References

[B1] Almeida da Silva RM Crespi AL (2013) *Scilla* L. In: Rico E Crespo MB Quintanar A Herrero A Aedo C (Eds) Flora iberica XX (Liliaceae–Agavaceae).CSIC, Madrid, 145–156.

[B2] Bachman S Moat J Hill AW De La Torre J Scott B (2011) Supporting Red List threat assessments with GeoCAT: Geospatial conservation assessment tool. ZooKeys 150: 117–126. 10.3897/zookeys.150.2109 PMC3234434 22207809

[B3] Blanca G Carmona R Cueto M Fuentes J (2023) *Linariapseudamethystea* (Antirrhineae, Plantaginaceae), a new species mimetic of and apparently sympatric with *L.amethystea*. Phytotaxa 585(1): 1–18. 10.11646/phytotaxa.585.1.1

[B4] Brotero FA (1828) Phytographia Lusitaniæ selectior, vol. 2. Ex Typographia Regia, Olisipone [Lisbon], 263 pp. https://bibdigital.rjb.csic.es/idurl/1/9668

[B5] Burbrink FT Crother BI Murray CM Smith BT Ruane S Myers EA Pyron RA (2022) Empirical and philosophical problems with the subspecies rank. Ecology and Evolution 12(7): e9069. 10.1002/ece3.9069 PMC9271888 35845367

[B6] Carapeto A (2020) Península do Ancão e pinhais do Garrão. In: Porto M (Ed.) Sítios de Interesse Botânico de Portugal Continental.Imprensa Nacional, Lisboa, 142–153.

[B7] Carapeto A Francisco A Pereira P Porto M (2020) Lista Vermelha da Flora Vascular de Portugal Continental, Coleção «Botânica em Português», Volume 7. Lisboa, Imprensa Nacional, 372 pp. https://listavermelha-flora.pt/wp-content/uploads/2020/10/Lista_Vermelha_Flora_Vascular_Portugal_Continental_2020_versao_digital.pdf

[B8] Costa JC Lousã M Espírito Santo MD (1996) A vegetação do Parque Natural da Ria Formosa (Algarve, Portugal). Studia Botanica 15: 69–157.

[B9] Coutinho AXP (1906) As escrofulariáceas de Portugal. Boletim da Sociedade Broteriana (série 1) 22: 114–213.

[B10] Coutinho AXP (1916) Notas da Flora de Portugal III. Livrarias Aillaud e Bertrand, Paris-Lisboa, 12 pp.

[B11] Coutinho AXP (1935) Suplemento da Flora de Portugal. Boletim da Sociedade Broteriana (série 2) 10: 156.

[B12] Coutinho AXP (1939) Flora de Portugal (plantas vasculares) disposta em chaves dicotómicas, 2ª edição. Bertrand (Irmãos), Lisboa, 938 pp.

[B13] Domingues de Almeida J Araújo PV Clamote F Porto M Carapeto A Chozas S Pereira AJ Gomes CT (2024) *Linariaspartea* (L.) Chaz. – mapa de distribuição. Flora-On: Flora de Portugal Interactiva, Sociedade Portuguesa de Botânica. http://www.flora-on.pt/#wLinaria+spartea [accessed 03.02.2024]

[B14] Fernández-Mazuecos M Blanco-Pastor JL Gómez JM Vargas P (2013) Corolla morphology influences diversification rates in bifid toadflaxes (Linariasect.Versicolores). Annals of Botany 112(9): 1705–1722. 10.1093/aob/mct214 24142920 PMC3838546

[B15] Fernández-Mazuecos M Ferrer-Gallego PP Miguel M Glover BJ Sáez L (2018a) A synopsis of the Iberian clade of Linariasubsect.Versicolores (Antirrhineae, Plantaginaceae) based on integrative taxonomy. Plant Systematics and Evolution 304(7): 871–884. 10.1007/s00606-018-1517-0

[B16] Fernández-Mazuecos M Mellers G Vigalondo B Sáez L Vargas P Glover BJ (2018b) Resolving recent plant radiations: Power and robustness of genotyping-by-sequencing. Systematic Biology 67(2): 250–268. 10.1093/sysbio/syx062 28973686

[B17] Fritz U Ihlow F (2022) Citizen Science, taxonomy and grass snakes: iNaturalist helps to clarify variation of coloration and pattern in *Natrixnatrix* subspecies. Vertebrate Zoology 72: 533–549. 10.3897/vz.72.e87426

[B18] Gallego MJ (2005) *Xolantha* Raf. [*Tuberaria* (Dunal) Spach] In: Castroviejo S Aedo C Cirujano S Laínz M Montserrat P Morales R Muñoz Garmendia F Navarro C Paiva J Soriano M (Eds) Flora iberica III (Plumbaginaceae (partim)–Capparaceae).CSIC, Madrid, 351–365.

[B19] GBIF (2024) GBIF Home Page. https://www.gbif.org [accessed 31.01.2024]

[B20] Gomes Oliveira N (2015) A Flore Portugaise e as viagens em Portugal de Hoffmannsegg e link (1795–1801). Chiado Editora, Lisboa, 492 pp.

[B21] Henriques JA (1889) Flora Lusitanica Exsiccata: Centuriae VII et VIII. *Boletim da Sociedade Broteriana* (série 1) 7: 101.

[B22] Hoffmannsegg JC Link HF (1811) Flore Portugaise, Tome II(7). Charles Fréderic Amelang, Berlin.

[B23] iNaturalist (2024) iNaturalist. https://www.inaturalist.org [accessed 31.01.2024]

[B24] IUCN Standards and Petitions Committee (2022) Guidelines for Using the IUCN Red List Categories and Criteria. Version 15.1. Prepared by the Standards and Petitions Committee. https://www.iucnredlist.org/documents/RedListGuidelines.pdf

[B25] Medina L Aedo C (2022) Vascular Plants from the Journey through Portugal (1797–1801) by Hoffmannsegg and Link at the Herbarium of the Real Jardín Botánico of Madrid. Plants 11(18): 2438. 10.3390/plants11182438 36145838 PMC9501163

[B26] Moura D Boski T (1994) Ludo Formation – a new lithostratigraphic unit in Quaternary of Central Algarve. Gaia. Revista de Geociências 9: 41–47.

[B27] Nunes VL Mendes R Quartau JA Simões PC (2014) Current distribution raises concerns on the conservation of *Tettigettalnamariae* (Quartau & Boulard, 1995) (Hemiptera: Cicadoidea) in Portugal. Ecologi 7: 50–57.

[B28] Pereira M Rodilla JM Silva L Alves H (2007) *Cistuslibanotis* L. (Cistaceae) en el sur de Portugal: Ecología, fitosociología y fitoquímica. Studia Botanica 26: 89–102.

[B29] Pereira AJ Francisco A Porto M (2016) Flora-On: Occurrence data of the vascular flora of mainland Portugal. PhytoKeys 69: 105–119. 10.3897/phytokeys.69.9432 PMC5029143 27698587

[B30] POWO (2024) Plants of the World Online. Facilitated by the Royal Botanic Gardens, Kew. http://www.plantsoftheworldonline.org/ [accessed 03.02.2024]

[B31] Sáez L (2009) Linariasect.Versicolores (Benth.) Wettst. In: Castroviejo S Herrero A Benedí C Rico E Güemes J (Eds) Flora iberica XIII (Plantaginaceae–Scrophulariaceae).CSIC, Madrid, 311–322.

[B32] Sampaio G (1913) Lista das espécies representadas no herbário português: pteridófitas e spermáfitas. Tipografia Costa Carregal, Porto, 148 pp.

[B33] Sampaio G (1946) Flora Portuguesa, 2ª Edição. Imprensa Moderna, Porto, 792 pp.

[B34] Sutton DA (1988) A revision of the tribe Antirrhineae. Oxford University Press, Oxford, 575 pp. Thiers B (continuously updated) Index Herbariorum: a global directory of public herbaria and associated staff. New York Botanical Garden’s Virtual Herbarium. https://sweetgum.nybg.org/science/ih/ [accessed 09.03.2024]

[B35] Viano J (1973) Résultats caryologiques de quelques espèces de *Linaria* et *Chaenorrhinum* récoltées au sud de la Péninsule Ibérique. Boletim da Sociedade Broteriana (série 2) 47: 323–331.

[B36] Viano J (1976) Les linaires à graines aptères du Bassin méditerranéen occidental. PhD Thesis, Université Aix-Marseiile III, France.

[B37] Viano J (1978) Les linaires à graines aptères du bassin méditerranéen occidental. 1. Linariasect.Versicolores. Candollea 33: 33–88.

[B38] Vigalondo B Fernández-Mazuecos M Vargas P Sáez L (2015) Unmasking cryptic species: Morphometric and phylogenetic analyses of the Ibero-North African *Linariaincarnata* complex. Botanical Journal of the Linnean Society 177(3): 395–417. 10.1111/boj.12251

[B39] Willkomm HM Lange JMC (1861) Prodromus florae Hispanicae, vol. 2. Sumtibus E. Schweizerbart (E. Koch), Stuttgartiae, 680 pp.

